# The synergistic effect of dual use of amphetamine-type stimulants and ketamine on drug-induced psychotic symptoms in Chinese synthetic drug users

**DOI:** 10.18632/oncotarget.16474

**Published:** 2017-03-22

**Authors:** Xue-Bing Liu, Yao Zhang, Xu-Yi Wang, Wei Hao

**Affiliations:** ^1^ Mental Health Institute of the Second Xiangya Hospital, Central South University, The China National Clinical Research Center for Mental Health Disorders, National Technology Institute of Psychiatry, Key Laboratory of Psychiatry and Mental Health of Hunan Province, Changsha, Hunan, China; ^2^ Affiliated Wuhan Mental Health Center, The Ninth Clinical School, Tongji Medical College of Huazhong University of Science & Technology, Wuhan, Hubei, China; ^3^ Wuhan Wudong Hospital, Wuhan Second Mental Hospital, Wuhan, Hubei, China

**Keywords:** amphetamine-type stimulants, ketamine, psychotic symptoms, synergistic effect

## Abstract

The use of amphetamine-type stimulants (ATS) and ketamine is of particular clinical concern because of its associated psychotic symptoms. In Chinese clinical practice, ATS and ketamine are commonly used simultaneously, but very few studies have reported the symptom profile of users who use both drugs. This study determined whether the combined use of ATS and ketamine is associated with more psychotic symptoms than either ATS or ketamine alone. According to drug use characteristics, 375 Chinese synthetic drug users were categorized into 2 pairs of comparison groups: ATS-only (*n*=125) *vs*. ATS-mainly (ATS most of the time and ketamine sometimes, *n*=150) and ketamine-only (*n*=38) *vs*. ketamine-mainly (ketamine most of the time and ATS sometimes, *n*=62). We used the Chinese Brief Psychiatric Rating Scale (BPRS) to assess these patients’ psychotic symptoms. ATS-mainly group had more anxiety/depression and anergia symptoms than ATS-only group (*p*<0.001), and ketamine-mainly group had more thinking-disorder, activity and hostility-suspicion symptoms than ketamine-only group (*p*≤0.001). These findings indicate that ATS may exacerbate the thinking-disorders, activity and hostility-suspicion symptoms of ketamine users, and ketamine may exacerbate anxiety/depression and anergia symptoms of ATS users.

## INTRODUCTION

Over the past 2 decades, amphetamine-type stimulants (ATS) and ketamine have become increasingly prevalent in China [1-3]. According to China’s Annual Report on Drug Control issued by the Narcotic Control Commission, the number of illicit drug users, who had been registered in the Department of Public Security, was 24.75 million in 2013; among them, synthetic drug users accounted for almost a half [4]. ATS and/or ketamine dependence and/or abuse is of particularly clinical concern, because of its associated psychotic symptoms [5-8]. Recent studies reported that, 26-46% of individuals who use ATS or ketamine experienced psychotic symptoms [9], which occurred more frequently than cocaine [10] and cannabis users [11].

Previous research indicates that chronic/heavy ATS or ketamine use may induce psychotic symptoms resembling positive symptoms of schizophrenia, such as suspiciousness, delusions of persecution and hallucinations [12, 13], perceptual changes, ideas of reference, thought disorder [14-16] , and some negative symptoms like blunted affect and diminished spontaneity [17, 18]. In China, one recent study reported more psychiatric symptoms and more severe drug-induced schizophrenia-like symptoms in chronic methamphetamine users than chronic ketamine users [19]. However, findings from this study may have limited generality, because ATS and ketamine are often used together in China, which may lead to additional or more severe psychotic symptoms [20]. For example, one study conducted in the United States reported that ATS and ketamine taken together resulted in additive effects, leading to thought disorder, arousal, euphoria and less-than-additive effects on psychosis [21]. To the best of our knowledge, few clinical studies had investigated the synergistic effect of dual use of ATS and ketamine on drug-induced psychotic symptoms in China. Given the increasing number of users of ATS and ketamine in China, it is important to understand the effects of combination use on psychotic symptoms of synthetic drug users.

The current study examined psychotic symptoms among adult inpatients who diagnosed with ATS and/or Ketamine induced psychosis. To determine the synthetic effects of combined ATS and ketamine use on users’ psychiatric symptom profiles, we compared psychotic symptoms between patients who use both ATS and ketamine, and ATS or ketamine alone.

## RESULTS

### Basic characteristics of study participants

The majority of participants in our sample were young males with low education levels and unstable jobs. Detailed demographic and drug use characteristics for the 4 groups were shown in Table 1 and Table 2. There were no statistically significant differences on demographic and drug use characteristics between the ATS-mainly group and the ATS-only group, and the ketamine-mainly group and the ketamine-only group.

**Table 1:** Demographic characteristics of study participants

Variables	AST mainly (*n*=150)	ATS only (*n*=125)	*P*	K mainly (*n*=62)	K only (*n*=38)	*P*
Gender						
Male	125(83.3)	99 (79.2)	0.380	49(79.0)	30 (78.9)	0.591
Female	25(16.7)	26 (20.8)		13(21.0)	8 (21.1)	
Age						
≤20	5(3.3)	2 (1.6)	0.219	3(4.8)	3 (7.9)	0.487
21-30	76(50.7)	68 (54.4)		33(53.2)	19 (50.0)	
1-40	51(34.0)	48 (38.4)		20(32.3)	15 (39.5)	
>41	18(12.0)	7 (5.6)		6(9.7)	1 (2.6)	
Marital Status						
Single	68(45.3)	48 (38.4)	0.474	28(45.2)	21 (55.3)	0.107
Married	64(42.7)	62 (49.6)		24(38.7)	16 (42.1)	
Divorced/Separated	18(12.0)	15 (12.0)		10(16.1)	1 (2.6)	
Education levels						
Less 9 years	126(84.0)	94 (75.2)	0.138	52(83.9)	28 (73.7)	0.206
High school	20(13.3)	23 (18.4)		5(8.1)	7 (18.4)	
College or above	4(2.7)	8 (6.4)		5(8.1)	3 (7.9)	
Occupation						
Unemployed	52(34.7)	17 (13.6)	<0.001	19(30.6)	6 (15.8)	0.409
Businessman	37(24.7)	41 (32.8)		12(19.4)	10 (26.3)	
Entertainment	11(7.3)	6 (4.8)		6(9.7)	4 (10.5)	
Migrant worker	50(33.3)	61 (48.8)		25(40.3)	18 (47.4)	
Urine test						
ATS (+)	114	86	---	3	0	--
ATS (-)	36	39		59	0	
K (+)	40	0		58	17	
K (-)	110	0		4	21	

Note: Values are given as number (%).

**Table 2:** Drug use characteristics of study participants

Variables	ATS mainly (*n*=150)	ATS only (*n*=150)	*P*	K mainly (*n*=62)	K only (*n*=38)	*P*
ATS						
Age of first use	20.32±6.12	17.28±5.44	0.185	21.15±7.69		
Range, yrs	15-42	13-36		17-40		
Duration, months	44.20±13.27	42.32±11.88	0.327	18.41±4.72		
Range, months	12-96	6-102		9-36		
Frequency, per month	16.11±6.44	17.12±4.10	0.43	3.78±2.98		
Range, per month	10-28	8-30		1-6		
Single dose, g	0.45±0.43	0.47±0.32	0.114	0.28±0.34		
Range, g	0.1-0.2	0.1-1.0		0.05-0.3		
Ketamine						
Age of first use	18.13±8.66			20.13±8.19	21.67±10.13	0.560
Range,yrs	12-36			16-45	18-51	
Duration,months	52.35±15.40			62.92±18.86	65.06±17.38	0.384
Range,months	30-108			42-120	38-114	
Frequency,per month	3.21±2.14			10.19±4.87	10.42±4.13	0.782
Range,per month	1-6			4-14	2-16	
Single dose,g	0.35±0.32			0.42±0.34	0.45±0.42	0.445
Range,g	0.1-0.3			0.2-0.6	0.1-0.8	

Note: Values given as mean and standard deviations.

### The association between combined use of ATS and ketamine and psychotic symptoms

As displayed in Table 3, ATS-mainly group had more anxiety/depression and anergia symptoms than ATS-only group (*p* < 0.001), and ketamine-mainly group had more thinking-disorder, activity and hostility-suspicion symptoms than ketamine-only group (*P* ≤ 0.001). Overall, while there was no significant difference in BPRS total score between ATS-mainly and ATS-only groups (*P* = 0.217), ketamine-mainly group scored significantly higher on BPRS than ketamine-only group (*P* ≤ 0.001).

**Table 3:** Comparisons of BPRS scores between subjects of different drug use characteristics

BPRS	ATS mainly	ATS only	t	*P*	K mainly	K only	t	*P*
Anxiety/depression	1.93±0.84	1.53±0.35	4.975	<0.001	3.18±0.76	2.91±0.49	1.953	0.053
Anergia	1.30±0.30	1.06±0.11	8.48	<0.001	1.85±0.55	1.91±0.61	0.508	0.613
Thinking-disorder	3.00±0.80	3.15±0.71	1.629	0.105	2.16±0.62	1.74±0.57	3.389	0.001
Activity	2.97±0.66	3.06±0.68	1.111	0.267	2.40±0.82	1.61±0.32	5.671	<0.001
Hostility-suspicion	4.62±0.82	4.81±0.95	1.78	0.076	3.51±1.05	3.02±0.79	2.477	0.015
Total score	47.60±8.14	46.50±6.24	1.238	0.217	46.52±5.9	40.13±7.68	4.679	<0.001

## DISCUSSION

In this study, we found that the anxiety/depression and anergia symptom scores of ATS-mainly group were significantly higher than ATS-only group, while the total BPRS score, thought disorder, activity score and hostility-suspicion symptoms of ketamine-mainly group were more prevalent than ketamine-only group. These results suggest that the psychotic symptoms induced by the combined use of ATS and ketamine are more severe than those induced by ATS/ ketamine alone. Our data indicate that ketamine use may exacerbate depression and anergia symptoms of ATS users and, ATS may exacerbate the thinking-disorders, activity and hostility-suspicion symptoms of ketamine users.

Consistent with 2 earlier studies [21, 22], our findings suggest that the psychotic symptoms caused by the combination of ATS and ketamine are not the supra-additive or even fully additive psychotic effects of either ATS or ketamine alone. Previous studies have reported that the noncompetitive glutamate N-methyl-D-aspartate (NMDA) antagonist could regulate the dopamine release in nucleus accumbens [23-25]. Based on the dopamine hypothesis on mechanisms of psychotic symptoms of schizophrenia [26, 27] , it seems that ATS and ketamine may share similar psychopathological mechanism, which results in a ceiling effect on psychotic symptoms. However, findings from our study were a little different from this hypothesis, that is, we found that ATS-mainly group had significantly more symptoms of depression and anergia than the ATS-only, whereas ketamine-mainly group had significant more symptoms of thought disorders and activity and hostility-suspicion. We, therefore, speculated that ATS and ketamine might still have different psychopathological mechanisms. These results were confirmed by our previous study, in which we found the ATS and ketamine user present with different profile of psychiatric symptoms [20]. It is possible that the interaction of ATS and ketamine, at the neurological level, affecting NMDA glutamate receptor and monoaminergic systems in the human brain, may antagonize some effects of the other drugs [28, 29] , or the dopaminergic response induced by ketamine treatment, in general, acts as a compensatory mechanism [30-33], which exaggerates or attenuates the emergence of psychotic symptoms. Further experimental research is needed to understand the potential mechanism of combined ATS and ketamine on neural system.

There are some methodological limitations in this study. Firstly, this was an observational study, and we were not able to control all possible confounding factors such as the dosage of drugs that influence psychotic symptoms. Secondly, our study participants were inpatients from a large psychiatric specialty hospital. Some of them were admitted involuntarily and therefore these drug users may tend to be more aggressive and offensive. Hence, findings from this study may not be generalizable to other clinical settings. Thirdly, another commonly used scale of psychotic symptoms, the Positive and Negative Syndrome Scale (PANSS), which is more comprehensive in assessing psychotic symptoms than BPRS [34], was not used in this study. We would have more findings on the symptom profile of drug users if we used PANSS in the current study. Fourth, psychiatrists who assessed the psychotic symptoms were not blinded to drug use status of subjects. Finally, our study did not collect data on the duration of psychotic symptoms, because these symptoms can be chronic, acute, or both. This is a potential confounding factor that may bias the synergistic effect of dual use of ATS and ketamine, as demonstrated in this study.

In summary, this study provides some evidence of psychotic symptomatology for the synergistic effect of dual use of ATS and ketamine. It might be due to the interactions of NMDA glutamate receptor and monoaminergic systems in the human brain. One of the potential clinical implications is that ATS and ketamine may have different effects on psychopathology. Further mechanism research is needed to clarify the interaction between amphetamine and ketamine.

## MATERIALS AND METHODS

### Participants

Between January 2012 and December 2013, a total of 375 synthetic drug users admitted to the Drug Abuse Treatment Inpatient Ward of Wuhan Mental Health Center, were consecutively included in this study. The inclusion criteria were (1) met the criteria for “mental disorders due to psychoactive substance use/abuse” based on ICD-10 [35, 36], and (2) substances used were ATS and/or ketamine. The exclusion criteria were (1) co-occurring use of heroin, cocaine, marijuana, and other illegal substances (excluding nicotine), (2) alcohol use disorders, and (3) major medical conditions. Two licensed senior psychiatrists conducted all diagnostic assessment and clinical interviews.

According to patients’ self-reported drug use patterns, we divided all patients into 2 pairs of comparison groups: ATS-only group (used ATS only) (*n* = 125) *vs*. ATS-mainly group (used ATS most of the time and ketamine sometimes, *n* = 150), and ketamine-only group (used ketamine only, *n* = 38) *vs*. ketamine-mainly group (used ATS most of the time and ketamine sometimes) (*n* = 62). This study protocol was approved by the Ethics Committee of Wuhan Mental Health Center. All participants gave written informed consent.

### Demographics and drug use history

As a routine clinical procedure, all patients were asked to provide demographic data (gender, age, education and occupation) and urine samples for urine toxicology tests. Enzyme multiplied immunoassay technique was used in urine tests to determine the types of drugs used, including ATS, ketamine, heroin, methadone, cocaine, cannabis, and benzodiazepines. Drug use history was reported by patients, and additionally verified by urine drug test results and their family members.

### Psychiatric symptom assessment

On the day of admission, the Chinese Brief Psychiatric Rating Scale (BPRS) [37, 38] was administered to assess the psychotic and behavioral symptoms of drug users. The BPRS contains 18 items scored on a seven-point scale (1 = not present to 7 = extremely severe). The Chinese BPRS is divided into 5 subscales: anxiety/depression, anergia, thinking-disorder, activity, and hostility-suspicion. In general, BPRS total score reflects the severity of psychopathology, while its subscale scores reflect the symptoms profiles of psychotic symptoms [39-41]. A high score is interpreted as having severe psychotic symptoms. The Chinese BPRS has been proved to reliable and valid to assess psychotic symptoms [40, 42].

### Statistical analyses

All statistical procedures were carried out by using SPSS 20.0 for Windows. Independent sample *t*-test was used to compare the symptom scores between patients used dual drugs and a single drug. Chi-square test was used to compare the group differences for category variables. The statistical significance level was set at *p* < 0.05 (two-sided).
